# The Prediction of Venous Thromboembolism Using Artificial Intelligence and Machine Learning in Lower Extremity Arthroplasty: A Systematic Review

**DOI:** 10.1016/j.artd.2025.101672

**Published:** 2025-03-29

**Authors:** Davood Dalil, Sina Esmaeili, Ehsan Safaee, Sajad Asgari, Nooshin Kejani

**Affiliations:** a Faculty of Medicine, Shahed University, Tehran, Iran; b Student Research Committee, Faculty of Medicine, Shahed University, Tehran, Iran; c Department of Biomedical Engineering, Science and Research Branch, Islamic Azad University, Tehran, Iran

**Keywords:** Artificial intelligence, Machine learning, Venous thromboembolism, Total hip arthroplasty, Total knee arthroplasty, Deep vein thrombosis

## Abstract

**Background:**

Venous thromboembolism (VTE), including deep vein thrombosis and pulmonary embolism, is a common and serious complication following lower extremity arthroplasty, such as total hip and knee arthroplasty. Due to the increasing number of these surgeries, accurately predicting VTE risk is crucial. Traditional clinical prediction models often fall short due to their complexity and limited accuracy.

**Methods:**

This Preferred Reporting Items for Systematic Review and Meta-Analyses–guided systematic review summarized the application of artificial intelligence (AI) and machine learning models in predicting VTE after total joint arthroplasty. Databases including PubMed, Scopus, Web of Science, and Embase were searched for relevant studies published up to January 2024. Eligible studies focused on the predictive accuracy of AI algorithms for VTE post arthroplasty and were assessed for quality using the Newcastle-Ottawa Scale.

**Results:**

A total of 7 retrospective cohort studies, encompassing 579,454 patients, met the inclusion criteria. These studies primarily employed the extreme gradient boosting model, which generally demonstrated strong predictive performance with area under the curve values ranging from 0.71 to 0.982. Models like random forest and support vector machines also performed well. However, only 1 study included external validation, critical for assessing generalizability.

**Conclusions:**

AI and machine learning models, particularly extreme gradient boosting, exhibit significant potential in predicting VTE after lower extremity arthroplasty, outperforming traditional clinical prediction tools. Yet, the need for external validation and high-quality, generalizable datasets remains critical before these models can be widely implemented in clinical practice. The study underscores the role of AI in preoperative planning to enhance patient outcomes in orthopaedic surgery.

## Introduction

Venous thromboembolism (VTE), including deep vein thrombosis (DVT) and pulmonary embolism (PE), is a common complication following lower limb arthroplasty including total hip arthroplasty (THA) and total knee arthroplasty (TKA). The incidence of VTE, comprising both DVT and PE, varies between 1% and 3% influenced by various factors [1,2]. With projections indicating an annual increase to 1.92 million TKA and 850,000 THA procedures in the United States by 2030, early diagnosis and treatment of VTE are becoming increasingly critical [3].

Currently, clinical practice uses various methods to predict VTE, such as measurement scales that are developed based on statistical analysis of clinical data. These scales include the Autar DVT scale [4], the JFK Medical Center DVT risk assessment tool [5], the Padua Prediction Score [6], the Risk Assessment Profile for Thromboembolism scale [7], and the Caprini Score for the prediction of VTE [8]. Among these, the Caprini score is the most widely used in the field of orthopaedics [9]. However, these scales often have certain limitations, including the considerable time and effort required for scoring, low accuracy in prediction, limited consideration of influencing factors, and the inability to provide real-time early warnings that adapt to changing conditions [10].

Recently, medicine has experienced remarkable advancements in technology, particularly in the application of artificial intelligence (AI), leading to significant changes in diagnostic and treatment approaches. This progress has spurred extensive research into AI-based prediction models for outcomes following total joint arthroplasty (TJA). These models are designed to aid in preoperative management, informed consent, shared decision-making, and reimbursement programs based on risk adjusting [[11], [12], [13]]. Despite these advancements, there remains a lack of accurate and validated risk prediction models specifically tailored for short-term outcomes of the TJA [13]. Thus, this study aimed to provide a thorough summary of the AI algorithms used for predicting the risk of VTE following TJA of the lower extremities.

## Material and methods

### Search strategy

This systematic review was conducted in accordance with the Preferred Reporting Items for Systematic Review and Meta-Analyses guidelines. The PubMed, Scopus, Web of Science, and Embase databases were systematically searched for published articles about the application of AI algorithms for predicting VTE following arthroplasty of lower extremities based on title, abstract, keywords, and medical subject headings. The following search terms were used to retrieve articles and abstracts: ("artificial intelligence" OR "deep learning" OR "machine learning" OR "computer-assisted diagnosis" OR "computer-assisted decision making" OR "data mining" OR "artificial neural networks") AND ("total hip arthroplasty" OR "total knee arthroplasty" OR "total ankle arthroplasty") AND ("venous thromboembolism" OR "embolism" OR "thromboembolism"). Our complete search strategy is available in the Supplementary File section.

### Eligibility criteria

The review screened for randomized controlled trials, and retrospective or prospective cohort studies. We limited our search to studies that were available in the English language and had to be published as full-text articles up to January 2024. Included studies required sufficient and relevant data related to at least one of the following criteria: the accuracy, specificity, and sensitivity of the AI algorithm to predict VTE following THA or TKA. No restrictions were set regarding the study or patient follow-up period.

We excluded studies that have at least one of these statements: non-AI or machine learning (ML) predictions, upper limb joint arthroplasty, nontotal or partial hip or knee arthroplasty, arthrodesis procedures, and studies reporting undesirable or unintentional postsurgical complaints such as fractures and joint dislocations. Additionally, we excluded review studies, case reports, congress abstracts, letters, and non-English publications.

### Data extraction

The following information was compiled by two independent authors for each study: author, publication date, country, study design, surgical procedure, database, follow-up time, study population’s data, type of VTE study metrics, ML model, and accuracy of VTE prediction.

### Assessment of study quality

The quality of all included studies was assessed by two independent reviewers using the Newcastle-Ottawa Scale (NOS), which evaluates studies based on three main criteria: the selection of study groups, the comparability of the groups, and the ascertainment of either exposure or outcome. Studies were then categorized into three quality tiers based on their scores: high quality (scores more than 6), medium quality (scores of 5 or 6), and low quality (scores less than 5) [14].

### Ethical considerations

This study was exempt from Institutional Review Board approval as it involved the review of published literature and did not include any human subjects.

## Results

### Article selection process

**Figure 1:**
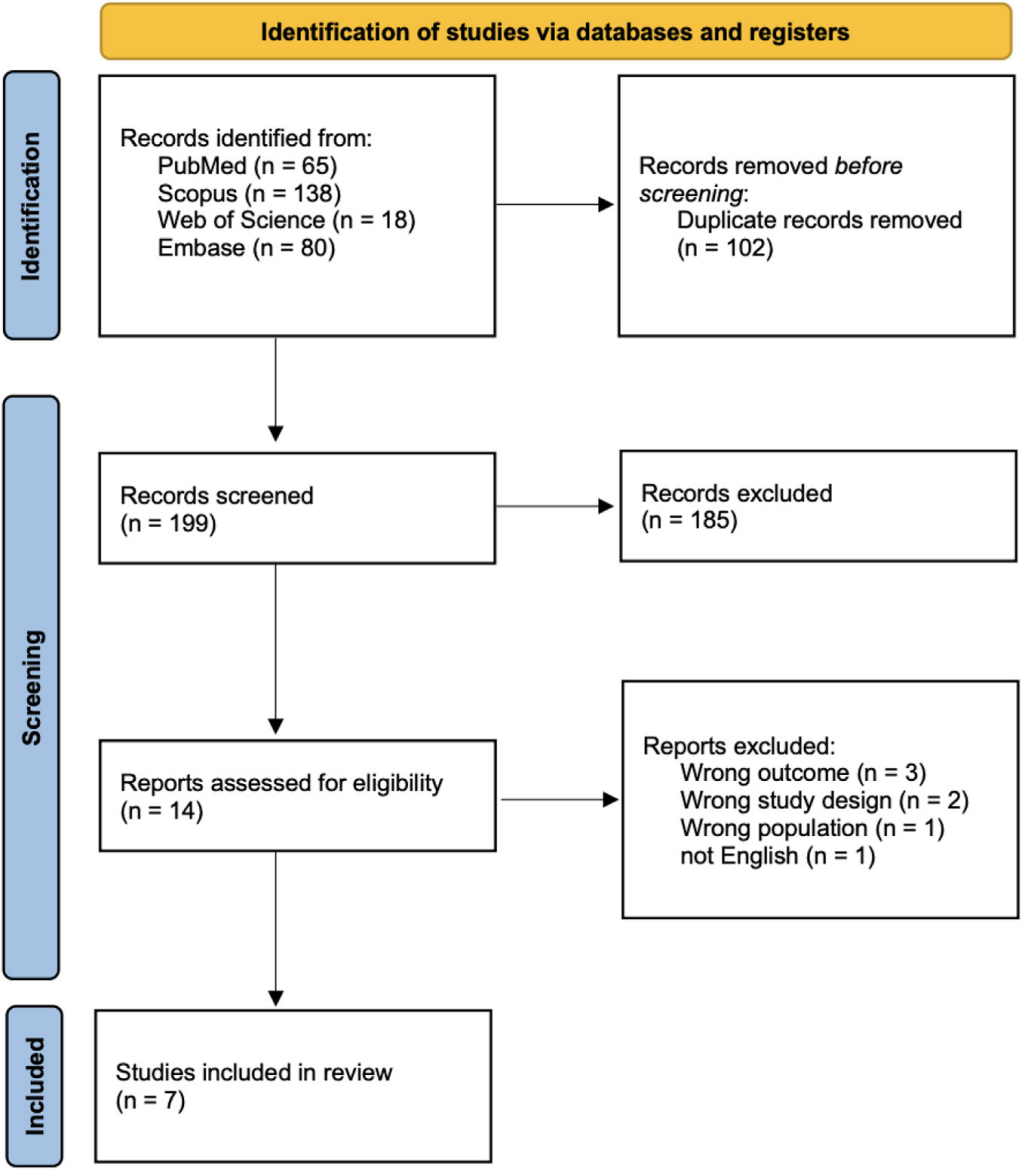
PRISMA 2020 flow diagram for new systematic reviews which included searches of databases and registers only. PRISMA, Preferred Reporting Items for Systematic Review and Meta-Analyses.

Following the removal of duplicate articles, we systematically reviewed 199 articles based on their titles and abstracts. Subsequently, 12 articles meeting our criteria were further assessed in their entirety. Articles not using ML models for the prediction of VTE were excluded, leaving us with a final selection of seven articles for inclusion in this systematic review [10,[15], [16], [17], [18], [19], [20]]. A visual representation of the article screening process is presented in Figure 1, following the Preferred Reporting Items for Systematic Review and Meta-Analyses guidelines.

### Study characteristics

The publication year of the studies ranged from 2019 to 2023. Collectively, these studies evaluated a total of 579,454 individuals. The average age across studies ranged from 55.9 to 77.6 years, while body mass index averaged between 22.94 and 32 kg/m^2^. Among the studies that reported gender distribution, 42% of patients were male. The geographical distribution of the studies consisted of 4 conducted in the United States [15,[18], [19], [20]] and 3 in China [10,16,17].

All the included studies were retrospective cohorts. Three studies focused on primary THA and TKA [10,18,19], 1 study examined revision THA and TKA [15], and 1 study encompassed both primary and revision THA and TKA [20]. Additionally, 2 studies were either primary TKA [16] or primary THA [17]. The duration of follow-up varied from 1 year to 11 years across the included studies. Five studies concentrated on VTE [15,[17], [18], [19], [20]], while 2 specifically examined DVT [10,16] (Table 1). Assessment of AI model predictive accuracy was carried out using metrics such as C-Statistics [15,18] and receiver operating characteristic curve [10,16,17,19,20] as detailed in the following sections.

**Table 1:** Characteristics of included studies.

Author (year)	Country	Study design	Population	Database	Surgery	Follow-up duration
Harris et al. (2019)	USA	Retrospective	107,792	ACS-NSQIP	Primary TKA & THA	2013-2014
Chen et al. (2022)	China	Retrospective	100	EHRs at The First Affiliated Hospital of Soochow University	Primary TKA	2019-2020
Abraham et al. (2022)	USA	Retrospective	34,056	NSQIP	Aseptic revision TKA & THA	2014-2019
Rasouli et al. (2022)	USA	Retrospective	392,661	Cerner Health Facts	Primary TKA & THA	2010-2015
Shohat et al. (2023)	USA	Retrospective	35,963	EHRs at Thomas Jefferson University	Primary & revision TKA & THA	2009-2020
Wang et al. (2023)	China	Retrospective	6897	EHRs at Peking University Third Hospital	Primary TKA & THA	2017-2021
Ding et al. (2023)	China	Retrospective	1985	EHRs at Shanghai Changzheng Hospital	Primary THA	2019-2021

ACS-NSQIP, American College of Surgeons-National Surgical Quality Improvement Program; EHRs, electronic health records; TKA, total knee arthroplasty; THA, total hip arthroplasty.

### Quality assessment of included studies

As per the assessment by NOS, the average score of the articles included in the analysis was 7.6 ± 1.2. With the exception of one study, all others were classified as possessing high methodological quality (NOS score ≥ 7). However, Chen et al’s study was categorized as having moderate methodological quality, primarily due to its lower comparability score (Table 2) [16].

**Table 2:** Quality assessment of included studies by Newcastle-Ottawa scale tool.

Author (Year)	Newcastle-Ottawa scale (NOS)			Total score
	Selection	Comparability	Outcome	
Harris et al. (2019)	∗∗∗∗	∗∗	∗∗∗	9/9
Abraham et al. (2022)	∗∗∗∗	∗∗	∗∗∗	9/9
Chen et al. (2022)	∗∗∗∗	/	∗∗	6/9
Rasouli et al. (2022)	∗∗∗∗	/	∗∗∗	7/9
Shohat et al. (2023)	∗∗∗∗	/	∗∗∗	7/9
Wang et al. (2023)	∗∗∗∗	∗	∗∗∗	8/9
Ding et al. (2023)	∗∗∗∗	/	∗∗∗	7/9

∗ represents a score of 1 point. ∗∗ represents a score of 2 points.

∗∗∗ represents a score of 3 points.

∗∗∗∗ represents a score of 4 points.

### Performance evaluation of machine learning models

Among the reviewed studies, 5 investigated the use of the extreme gradient boosting (XGB) model for predicting VTE/DVT [10,[15], [16], [17],^20^]. One study reported a C-statistics value of 0.56, indicating poor predictive performance [15]. However, other studies reported area under the curve (AUC) values ranging from 0.71 to 0.982 for the XGB model, suggesting favorable predictive ability. Notably, Shohat et al. examined multiple algorithms, including random forest, least absolute shrinkage and selection operator, and support vector machines, with XGB (AUC = PE: 0.80, DVT: 0.71) consistently demonstrating superior AUC values [20].

Similarly, Ding et al. observed a similar trend across 6 algorithms (AUC [95% confidence interval {CI}] = 0.982 [0.954-1.000]), including XGB, logistic regression, multilayer perceptron, adaptive boosting, gradient boosting tree model, and k-nearest neighbor [17]. In this study, the 6 investigated algorithms had AUC values between 0.837 and 0.982, whereas traditional scoring systems reported values ranging from 0.522 to 0.716 [17]. Moreover, in the study by Rasouli et al., this superiority over the traditional scoring system is evident [19]. Additionally, Wang et al. reported that all models exhibited AUC values more than 0.88, with the XGB model (AUC [95% CI] = 0.915 [0.894-0.935]) demonstrating the best performance following the Ensembled model (AUC [95% CI] = 0.921 [0.896-0.936]) [10].

Rasouli et al. [19], Shohat et al., and Wang et al. [10] investigated the random forest model, which produced AUC values ranging from 0.62 to 0.91. The logistic regression model was examined by Wang et al. [10] and Ding et al. [17], with reported AUC values and 95% CI of 0.885 (0.868-0.902) and 0.944 (0.878-1.000), respectively. Similarly, Wang et al. [10] and Shohat et al. [20] investigated the support vector machine model, with Wang et al. reporting superior performance (AUC [95% CI] = 0.903 [0.874-0.930]) compared to Shohat et al. (AUC for PE: 0.67, DVT: 0.64). Harris et al.’s [18] study revealed a C-statistics value and 95% CI of 0.613 (0.608-0.617) for the least absolute shrinkage and selection operator model, indicating low prediction accuracy, while Shohat et al. [20] reported AUC values of 0.8 for PE and 0.68 for DVT. Additional details on other models and study limitations are provided in Table 3.

**Table 3:** Evaluating the prediction accuracy of machine learning models.

Author (year)	Intervention	VTE	Groups/validation	Metrics (95% CI)	ML model	Statistical results	Outcome	Comparison	Limitations
Harris et al. (2019)	Prediction of 30-d mortality and complications	DVT or PE	Training or test not reported/internal validation: 107,792; external validation: 70,569	C-statistic	LASSO	0.613	The models demonstrated poor accuracy for VTE prediction.	None	1. ACS-NSQIP dataset is limited to sampled TJAs from participating hospitals. 2. Not containing complete information on comorbidities or other patient and setting factors.
Abraham et al. (2022)	Prediction of 30-d mortality and complications	DVT or PE	Training: 27,011; Test: 7045/internal validation, temporal validation but no external validation	C-statistic	XGB	0.59	The model demonstrated poor discrimination for VTE.	None	1. Retrospective design 2. Lack of external validation 3. Comorbidity severity not accounted 4. Surgeon-specific factors not considered 5. Calibration issues 6. Interpretability of the XGB model
Chen et al. (2022)	Prediction of DVT 3-5 d after surgery	DVT	Training: 60; Test: 40/internal validation but no external validation	AUC	XGB	0.832	The established model can predict the occurrence of DVT after TKA with good prediction performance	Conventional RA	1. Retrospective design 2. Small sample size 3. Not enough influencing factors in the analysis and no hierarchical analysis of these factors
Rasouli et al. (2022)	Risk assessment of VTE within 30 d of surgery	DVT or PE	Training: 274,863; Test: 117,798/internal validation but no external validation	AUC	GBT	0.638	Several new risk factors were identified for VTE. The best FCDNN model, trained with these selected features, achieved an AUC of 0.873, significantly outperforming models using only previously known risk factors.	None	The use of the obsolete ICD-9 standard for extracting diagnoses and medication predictors
					RF	0.698			
					TE	0.635			
					FCDNN	0.780			
Shohat et al. (2023)	Prediction of VTE and MBE within 90 d of surgery	DVT or PE	Training: 25,174; Test: 10,789/internal validation but no external validation	AUC	XGB	PE: 0.80 DVT: 0.71	XGB had the highest performance for both PE and DVT prediction.	Conventional RA	1. Retrospective design 2. Lack of interpretability 3. Underpowered analysis 4. Uncertain DVT classification 5. Inclusion of assumed variables 6. Limited assessment of variables
					RF	PE: 0.70 DVT: 0.62			
					LASSO	PE: 0.8 DVT: 0.68			
					SVM	PE: 0.67 DVT: 0.64			
Wang et al. (2023)	Prediction of DVT within 5 wks after surgery	DVT	Training: 5518; Test: 1379/internal validation but no external validation	AUC	XGB	0.915	The ensemble model had the highest AUC and F1 score, but combining models improve generalization and mitigate underfitting/overfitting.	Conventional RA	1. Retrospective study 2. Dependency on ultrasonographic diagnosis 3. Small sample size 4. Symptomatology of DVT patients 5. Risk of underfitting/overfitting
					RF	0.907			
					SVM	0.903			
					LR	0.885			
					BPNN	0.910			
					Ensembled	0.921			
Ding et al. (2023)	Prediction of DVT and PE within 30 d after surgery	DVT	Training: 1036; Test: 445/internal validation but no external validation	AUC	XGB	0.982	Developed tool showed strong predictive performance; XGB excelled with a 0.982 AUC and the highest net benefit on the DCA curve.	Conventional RA	1. Short-term outcome focus 2. Incomplete data on patient transfers 3. Uncertainty in outcome determination 4. Risk of underfitting/overfitting
					LR	0.944			
					MLP	0.955			
					GBC	0.978			
					KNN	0.837			
					AdaBoost	0.980			

ACS-NSQIP, American College of Surgeons-National Surgical Quality Improvement Program; AdaBoost, adaptive boosting; AUC, area under curve; BPNN, backpropagation neural network; CI, confidence interval; DCA, decision curve analysis; DVT, deep venous thrombosis; FCDNN, fully connected deep neural network; GBC, gradient boosting tree model; GBT, gradient boosted trees; ICD-9, International Classification of Diseases, Ninth Revision; KNN, k-nearest neighbor; LASSO, least absolute shrinkage and selection operator; LR, logistic regression; MBE, major bleeding event; MI, myocardial infarction; MLP, multilayer perceptron; PE, pulmonary embolism; RA, risk assessment; RF, random forest; SVM, support vector machine; TE, tree ensemble; TJA, total joint arthroplasty; TKA, total knee arthroplasty; VTE, venous thromboembolism; XGB, extreme gradient boosting.

### Factors associated with VTE

In the study by Chen et al., the most important features in the XGB model were identified as multiple injuries, time from injury to operation, age, coronary heart diseases, D-dimer levels 1 day postsurgery, and hypertension, respectively [16]. According to Shohat et al., key factors associated with PE included active cancer, hypercoagulopathy, blood transfusion, use of Warfarin for VTE prophylaxis, older age, operative duration, revision surgery, history of VTE, atrial fibrillation, and underlying fracture. For DVT, significant factors included hypercoagulopathy, older age, allogenic blood transfusions, revision surgery, Warfarin prophylaxis, simultaneous bilateral surgery, active cancer, active or former smoking, underlying fracture, and male sex [20].

## Discussion

To our knowledge, this systematic review is the first of its kind, identified a total of 7 studies, including a total of 23 ML models and data of 579,454 patients, evaluating the accuracy and reliability of AI/ML models in predicting VTE after TJA of lower extremities. Among all included studies, the XGB model, which uses a series of decision rules (decision trees) to analyze data and improve predictions iteratively [21], was the most commonly used ML model for predicting VTE after TJA (5 studies). While 1 study reported poor discrimination, the remaining studies demonstrated good to excellent prediction performance for DVT or PE, with AUC values ranging from 0.71 to 0.982. Following XGB, the adaptive boosting and Ensembled models predicted DVT with a very high AUC of 0.980 and 0.921, respectively. Notably, only 1 study performed external validation of its model. Five of the reviewed studies were focused on the prediction of DVT or PE, while 2 of them investigated the prediction of 30-d mortality and complications after arthroplasty. Considering specific VTE risk factors such as time from injury to operation, hemoglobin level, D-dimer level, hypercoagulability, VTE prophylaxis, and so on could explain the performance difference of the prediction models in the 5 studies focused on DVT or PE as an outcome with the other 2 studies.

In recent years, AI has come into the spotlight due to its potential to bring significant changes to various areas of the health sector, including disease diagnosis, management, and prognosis, and assessing the risk of complications. In terms of orthopaedic surgery, AI has been increasingly applied to knee and hip arthroplasties and its role in clinical decision-making, image interpretation, surgical planning, and prediction of postoperative complications and mortality has been increasingly evaluated. A systematic review by Lopez et al. showed the best performance of AI/ML models with an average AUC of 0.8 in predicting postoperative adverse effects, mortality, and patient-reported outcomes [22].

The other benefit of AI/ML predictive models is that they support health providers in creating and optimizing patient-specific needs and individualized surgical plans for patients undergoing lower extremity arthroplasties that mostly involve older populations with comorbidities. Precise identification of an implant for revision surgery and classification of TKA candidates based on personalized risk factors are such examples [[22], [23], [24]]. The application of AI for medical image analysis is a prominent area of research, and this trend extends to orthopaedics. Karnuta et al. demonstrated an excellent performance, with an AUC of 0.999, identifying TKA and THA implants from radiographs [25,26].

AI models can assist surgeons in predicting postoperative complications. Previously, several studies have demonstrated the application of AI/ML models to predict postoperative pain in patients at high risk for prolonged opioid administration after surgery [27,28]. VTE is one of the severe postsurgery complications with high morbidity and mortality. Thus, VTE risk stratification is of high importance for surgeons. By integrating various risk factors of VTE into big-data-trained AI models, our study showed that AI can help surgeons with individualized VTE risk prediction following TKA or THA.

AI/ML models have emerged as a powerful predictive tool in clinical decision-making due to their ability to integrate large amounts of data and find complex patterns. However, many factors still influence the model performance including the types of models used, dataset quality, limited sample size, and optimization methods. Providing high-quality data to train and test the ML models could be ensured by the help of clinicians in accurate data collection, annotation, and auditing. Models based on single-center datasets may lack generalization due to variations in variables or outputs as well as several biases including selection bias and misclassified diagnosis [29].

Furthermore, one cannot rely solely on internal validation as this may lead to false optimism by overestimating the AUC [30]. As a result, it is not possible to assess the generalizability of a model to other populations, such as people of different regions, ages, or insurance status. On the other hand, it has been demonstrated that external validation reveals a significantly worse performance of AI/ML predictive models [31,32]. This highlights the importance of external validation before any clinical translation of the model. Among the included studies in this review, just 1 study externally validated the model which showed a poor accuracy for VTE prediction.

This study has several limitations. First, all included studies were retrospective in nature, which inherently carries risks of selection bias and unmeasured confounders that may impact the reported predictive accuracy of AI models. Second, the heterogeneity in study populations, datasets, and evaluation metrics complicates direct comparisons of model performance. Finally, while this review provides valuable insights into AI-based VTE prediction, it does not address practical implementation challenges, such as the need for user-friendly interfaces, integration into clinical workflows, and potential ethical considerations related to data privacy and bias. Future studies with prospective designs and robust external validation are necessary to address these limitations and advance the clinical application of AI in VTE prediction.

Despite these limitations, this study systematically reviews the existing literature and highlights the potential of AI and ML models, particularly the XGB algorithm, to predict VTE for patients undergoing lower extremity arthroplasty. These models enable precise preoperative risk stratification, aiding healthcare providers in tailoring thromboprophylaxis and optimizing surgical planning. Early identification of high-risk patients can reduce VTE incidence through targeted monitoring and interventions, while AI-driven decision support systems streamline workflows by integrating patient data to enhance clinical outcomes.

## Conclusions

AI algorithms are increasingly applied in various fields of orthopaedics, particularly preoperative and postoperative management of TJA. Although these models have performed promising in predicting postoperative complications including VTE, clinical translation remains a challenge. Larger datasets and more advanced technologies should be applied to increase the accuracy of these predictive models. Furthermore, external validation of these AI/ML models must be proven to be generalizable. Future studies should address these challenges to develop these models into reliable tools for aiding physicians in clinical practice.

## Funding

The authors received no financial support for this study.

## Conflicts of interest

The authors declare there are no conflicts of interest.

For full disclosure statements refer to https://doi.org/10.1016/j.artd.2025.101672.

## Availability of data and materials

The present study was performed based on published literature and no datasets were generated.

## CRediT authorship contribution statement

**Davood Dalil:** Writing – review & editing, Supervision, Methodology, Formal analysis, Data curation, Conceptualization. **Sina Esmaeili:** Writing – original draft, Visualization, Validation, Supervision, Conceptualization. **Ehsan Safaee:** Writing – review & editing, Supervision, Project administration, Methodology, Formal analysis, Data curation, Conceptualization. **Sajad Asgari:** Writing – original draft, Project administration. **Nooshin Kejani:** Visualization, Data curation.

## Figures and Tables

**Table s1:** Complete search strategy.

Database	Search strategy
PubMed	#1 "Artificial Intelligence"[Mesh] OR "Artificial Intelligence"[Title/Abstract] OR "Deep Learning"[Mesh] OR "Deep Learning"[Title/Abstract] OR "Machine Learning"[Mesh] OR "Machine Learning"[Title/Abstract] OR "Machine learning prediction"[Title/Abstract] OR "Artificial neural networks"[Title/Abstract] OR "Diagnosis, Computer-Assisted"[Mesh] OR "Diagnosis, Computer-Assisted"[Title/Abstract] OR "Decision Making, Computer-Assisted"[Mesh] OR "Decision Making, Computer-Assisted"[Title/Abstract] OR "Data Mining"[Mesh] OR "Data Mining"[Title/Abstract] OR "Algorithms"[Mesh] OR "Algorithms"[Title/Abstract] #2 "Arthroplasty, Replacement, Hip"[Mesh] OR "Arthroplasty, Replacement, Hip"[Title/Abstract] OR "Hip Prosthesis Implantation"[Title/Abstract] OR "Total Hip Replacement"[Title/Abstract] OR "hip replacement arthroplasty"[Title/Abstract] OR "total hip arthroplasty"[Title/Abstract] OR "hip replacement"[Title/Abstract] OR "Hip Prosthesis"[Mesh] OR "Hip Prostheses"[Title/Abstract] OR "Hip Prosthesis"[Title/Abstract] OR "Femoral Head Prosthesis"[Title/Abstract] OR THA[Title/Abstract] OR THR[Title/Abstract] #3 "Arthroplasty, Replacement, Knee"[Mesh] OR "Arthroplasty, Replacement, Knee"[Title/Abstract] OR "Knee Prosthesis Implantation"[Title/Abstract] OR "Total Knee Replacement"[Title/Abstract] OR "Knee replacement arthroplasty"[Title/Abstract] OR "total Knee arthroplasty"[Title/Abstract] OR "Knee replacement"[Title/Abstract] OR "Knee Prosthesis"[Mesh] OR "Knee Prostheses"[Title/Abstract] OR "Knee Prosthesis"[Title/Abstract] OR TKA[Title/Abstract] OR (TKR[Title/Abstract] OR "partial knee arthroplasty"[Title/Abstract] OR "unicompartmental knee arthroplasty"[Title/Abstract] OR "unicondylar knee arthroplasty"[Title/Abstract] OR "partial knee replacement"[Title/Abstract] OR "unicompartmental knee replacement"[Title/Abstract] OR "unicondylar knee replacement"[Title/Abstract] #4 "Arthroplasty, Replacement, Ankle"[Mesh] OR "Arthroplasty, Replacement, Ankle"[Title/Abstract] OR "Total Ankle Replacement"[Title/Abstract] OR "Ankle replacement arthroplasty"[Title/Abstract] OR "total Ankle arthroplasty"[Title/Abstract] OR "Ankle replacement"[Title/Abstract] OR TAA[Title/Abstract] OR TAR[Title/Abstract] #5 "Thromboembolism"[Mesh] OR "Thromboembolism"[Title/Abstract] OR "Embolism"[mesh] OR "Embolism"[Title/Abstract] OR "Embolus"[Title/Abstract] OR "Embolism, Paradoxical"[Mesh] OR "Embolism, Paradoxical"[Title/Abstract] OR "Embolism and Thrombosis"[Mesh] OR "Embolism and Thrombosis"[Title/Abstract] OR "Crossed Embolism"[Title/Abstract] OR "Thrombosis"[Title/Abstract] OR "Thrombosis"[Mesh] OR "Blood Clots"[Title/Abstract] OR "Venous Thromboembolism"[Mesh] OR "Venous Thromboembolism"[Title/Abstract] OR "venothromboembolism"[Title/Abstract] OR "thromboembolic"[Title/Abstract] OR DVT[Title/Abstract] OR VTE[Title/Abstract] OR PE[Title/Abstract] #6 #2 OR #3 OR #4 #7 #1 AND #6 AND #5
Scopus	#1 TITLE-ABS-KEY (“artificial intelligence”) OR TITLE-ABS-KEY (“deep learning”) OR TITLE-ABSKEY (“machine learning”) OR TITLE-ABS-KEY (“machine learning prediction”) OR TITLE-ABSKEY (“artificial neural networks”) OR TITLE-ABS-KEY (“diagnosis, computer-assisted”) OR TITLE-ABS-KEY (“decision making, computer-assisted”) OR TITLE-ABS-KEY (“data mining”) OR TITLE-ABS-KEY (“algorithms”) #2 TITLE-ABS-KEY (“arthroplasty, replacement, hip”) OR TITLE-ABS-KEY (“hip prosthesis implantation”) OR TITLE-ABS-KEY (“total hip replacement”) OR TITLE-ABS-KEY (“hip replacement arthroplasty”) OR TITLE-ABS-KEY (“total hip arthroplasty”) OR TITLE-ABS-KEY (“hip replacement”) OR TITLE-ABS-KEY (“hip prosthesis”) OR TITLE-ABS-KEY (“hip prostheses”) OR TITLE-ABS-KEY (“femoral head prosthesis”) OR TITLE-ABS-KEY (tha) OR TITLE-ABS-KEY (thr) #3 TITLE-ABS-KEY (“arthroplasty, replacement, knee”) OR TITLE-ABS-KEY (“knee prosthesis implantation”) OR TITLE-ABS-KEY (“total knee replacement”) OR TITLE-ABS-KEY (“knee replacement arthroplasty”) OR TITLE-ABS-KEY (“total knee arthroplasty”) OR TITLE-ABS-KEY (“knee replacement”) OR TITLE-ABS-KEY (“knee prosthesis”) OR TITLE-ABS-KEY (“knee prostheses”) OR TITLE-ABS-KEY (tka) OR TITLE-ABS-KEY (tkr) OR TITLE-ABS-KEY (“partial knee arthroplasty”) OR TITLE-ABS-KEY (“unicompartmental knee arthroplasty”) OR TITLE-ABS-KEY (“unicondylar knee arthroplasty”) OR TITLE-ABS-KEY (“partial knee replacement”) OR TITLEABS-KEY (“unicompartmental knee replacement”) OR TITLE-ABS-KEY (“unicondylar knee replacement”) #4 TITLE-ABS-KEY (“arthroplasty, replacement, ankle”) OR TITLE-ABS-KEY (“total ankle replacement”) OR TITLE-ABS-KEY (“ankle replacement arthroplasty”) OR TITLE-ABS-KEY (“total ankle arthroplasty”) OR TITLE-ABS-KEY (“ankle replacement”) OR TITLE-ABS-KEY (taa) OR TITLE-ABS-KEY (tar) #5 TITLE-ABS-KEY (“thromboembolism”) OR TITLE-ABS-KEY (“embolism”) OR TITLE-ABS-KEY (“embolus”) OR TITLE-ABS-KEY (“embolism, paradoxical”) OR TITLE-ABS-KEY (“embolism and thrombosis”) OR TITLE-ABS-KEY (“crossed embolism”) OR TITLE-ABS-KEY (“thrombosis”) OR TITLE-ABS-KEY (“blood clots”) OR TITLE-ABS-KEY (“venous thromboembolism”) OR TITLE-ABS- KEY (“venous thromboembolism”) OR TITLE-ABS-KEY (“venothromboembolism”) OR TITLEABS-KEY (“thromboembolic”) OR TITLE-ABS-KEY (dvt) OR TITLE-ABS-KEY (vte) OR TITLE-ABSKEY (pe) #6 #2 OR #3 OR #4 #7 #1 AND #5 AND #6
Web of Science	#1 TS=(("Artificial Intelligence") OR ("Deep Learning") OR ("Machine Learning") OR ("Machine learning prediction") OR ("Artificial neural networks") OR ("Diagnosis, Computer-Assisted") OR ("Decision Making, Computer-Assisted") OR ("Data Mining") OR ("Algorithms")) #2 TS=(("Arthroplasty, Replacement, Hip") OR ("Hip Prosthesis Implantation") OR ("Total Hip Replacement") OR ("hip replacement arthroplasty") OR ("total hip arthroplasty") OR ("hip replacement") OR ("Hip Prosthesis") OR ("Hip Prostheses") OR ("Femoral Head Prosthesis") OR (THA) OR (THR)) #3 TS=(("Arthroplasty, Replacement, Knee") OR ("Knee Prosthesis Implantation") OR ("Total Knee Replacement") OR ("Knee replacement arthroplasty") OR ("total Knee arthroplasty") OR ("Knee replacement") OR ("Knee Prosthesis") OR ("Knee Prostheses") OR (TKA) OR (TKR) OR ("partial knee arthroplasty") OR ("unicompartmental knee arthroplasty") OR ("unicondylar knee arthroplasty") OR ("partial knee replacement") OR ("unicompartmental knee replacement") OR ("unicondylar knee replacement")) #4 TS=(("Arthroplasty, Replacement, Ankle") OR ("Total Ankle Replacement") OR ("Ankle replacement arthroplasty") OR ("total Ankle arthroplasty") OR ("Ankle replacement")) OR (TAA) OR (TAR)) #5 TS=(("Thromboembolism") OR ("Embolism") OR ("Embolus") OR ("Embolism, Paradoxical")) OR ("Embolism and Thrombosis") OR ("Crossed Embolism") OR ("Thrombosis") OR ("Blood Clots") OR ("Venous Thromboembolism") OR ("Venous Thromboembolism") OR ("venothromboembolism") OR ("thromboembolic") OR (DVT) OR (VTE) OR (PE)) #6 #4 OR #3 OR #2 #7 #1 AND #5 AND #6
Embase	#1 ‘Artificial Intelligence’:ti,ab,kw OR ‘Deep Learning’:ti,ab,kw OR ‘Machine Learning’:ti,ab,kw OR ‘Computer Assisted’:ti,ab,kw OR ‘Data Mining’:ti,ab,kw OR ‘Algorithm’:ti,ab,kw #2 ‘artificial intelligence’/exp OR ‘machine learning’/exp OR ‘computer assisted diagnosis’/exp #3 #1 OR #2 #4 ‘hip prosthesis implantation’:ti,ab,kw OR ‘hip replacement arthroplasty’:ti,ab,kw OR ‘total hip replacement’:ti,ab,kw OR ‘total hip arthroplasty’:ti,ab,kw OR ‘total joint arthroplasty’:ti,ab,kw OR ‘total joint replacement’:ti,ab,kw OR THA:ti,ab,kw OR TJA:ti,ab,kw OR TJR:ti,ab,kw OR THR:ti,ab,kw OR ‘total hip replacement’/exp #5 ‘knee prosthesis implantation’:ti,ab,kw OR ‘knee replacement arthroplasty’:ti,ab,kw OR ‘total knee replacement’:ti,ab,kw OR ‘total knee arthroplasty’:ti,ab,kw OR TKA:ti,ab,kw OR TKR:ti,ab,kw OR ‘total knee arthroplasty’/exp #6 ‘Ankle prosthesis implantation’:ti,ab,kw OR ‘Ankle replacement arthroplasty’:ti,ab,kw OR ‘total Ankle replacement’:ti,ab,kw OR ‘total Ankle arthroplasty’:ti,ab,kw OR TAR:ti,ab,kw OR TAA:ti,ab,kw OR ‘total Ankle arthroplasty’/exp #7 #4 OR #5 OR #6 #8 ‘venous thromboembolism’/exp OR ‘Thromboembolism’:ti,ab,kw OR ‘Embolism’:ti,ab,kw OR ‘Embolus’:ti,ab,kw OR ‘Thrombosis’:ti,ab,kw OR ‘thromboembolic’:ti,ab,kw OR ‘venothromboembolism’:ti,ab,kw OR ‘Blood Clot’:ti,ab,kw OR ‘vte’:ti,ab,kw OR ‘dvt’:ti,ab,kw OR ‘pe’:ti,ab,kw #9 #3 AND #7 AND #8

## Appendix A Supplementary data

Conflict of Interest Statement for Asgari

Conflict of Interest Statement for Dalil

Conflict of Interest Statement for Esmaeili

Conflict of Interest Statement for Kejani

Conflict of Interest Statement for Safaee
